# Joint mapping of cardiovascular diseases: comparing the geographic patterns in incident acute myocardial infarction, stroke and atrial fibrillation, a Danish register-based cohort study 2014–15

**DOI:** 10.1186/s12942-021-00294-w

**Published:** 2021-08-30

**Authors:** Kristine Bihrmann, Gunnar Gislason, Mogens Lytken Larsen, Annette Kjær Ersbøll

**Affiliations:** 1 National Institute of Public Health, University of Southern Denmark, Studiestræde 6, 1455 Copenhagen, Denmark; 2 Department of Cardiology, The Cardiovascular Research Centre, Copenhagen University Hospital Herlev and Gentofte, Gentofte, Denmark; 3 Faculty of Health and Medical Sciences, Copenhagen University, Copenhagen, Denmark; 4 The Danish Heart Foundation, Copenhagen, Denmark; 5 Department of Clinical Medicine, Aalborg University, Aalborg, Denmark

**Keywords:** Cardiovascular disease, Geographic variation, Multiple-disease mapping, Epidemiology

## Abstract

**Background:**

Disease mapping aims at identifying geographic patterns in disease. This may provide a better understanding of disease aetiology and risk factors as well as enable targeted prevention and allocation of resources. Joint mapping of multiple diseases may lead to improved insights since e.g. similarities and differences between geographic patterns may reflect shared and disease-specific determinants of disease. The objective of this study was to compare the geographic patterns in incident acute myocardial infarction (AMI), stroke and atrial fibrillation (AF) using the unique, population-based Danish register data.

**Methods:**

Incident AMI, stroke and AF was modelled by a multivariate Poisson model including a disease-specific random effect of municipality modelled by a multivariate conditionally autoregressive (MCAR) structure. Analyses were adjusted for age, sex and income.

**Results:**

The study included 3.5 million adults contributing 6.8 million person-years. In total, 18,349 incident cases of AMI, 28,006 incident cases of stroke, and 39,040 incident cases of AF occurred. Estimated municipality-specific standardized incidence rates ranged from 0.76 to 1.35 for AMI, from 0.79 to 1.38 for stroke, and from 0.85 to 1.24 for AF. In all diseases, geographic variation with clusters of high or low risk of disease after adjustment was seen. The geographic patterns displayed overall similarities between the diseases, with stroke and AF having the strongest resemblances. The most notable difference was observed in Copenhagen (high risk of stroke and AF, low risk of AMI). AF showed the least geographic variation.

**Conclusion:**

Using multiple-disease mapping, this study adds to the results of previous studies by enabling joint evaluation and comparison of the geographic patterns in AMI, stroke and AF. The simultaneous mapping of diseases displayed similarities and differences in occurrence that are non-assessable in traditional single-disease mapping studies. In addition to reflecting the fact that AF is a strong risk factor for stroke, the results suggested that AMI, stroke and AF share some, but not all environmental risk factors after accounting for age, sex and income (indicator of lifestyle and health behaviour).

**Supplementary Information:**

The online version contains supplementary material available at 10.1186/s12942-021-00294-w.

## Background

Disease mapping aims at identifying geographic patterns in disease. This may provide a better understanding of disease aetiology and risk factors as well as enable targeted prevention and allocation of resources. Most commonly, disease mapping involves a single disease. However, joint modelling and mapping of multiple diseases may lead to improved insights since e.g., similarities and differences between geographic patterns may reflect shared and disease-specific determinants of disease that are non-assessable in traditional single-disease mapping studies. Moreover, similarities and differences between diseases may reflect more generic patterns that are not specific to the study area and may have broader implications than mapping a single disease.

The present study aimed at joint modelling and comparison of geographic patterns in three different cardiovascular diseases (CVD): Acute myocardial infarction (AMI), stroke and atrial fibrillation (AF). AMI and stroke are the most immediately serious conditions of the three. AF, however, is associated with increased risk of both AMI [1] and especially stroke [2]. Therefore, AF also represents a major public health problem. Overall, CVD is a leading cause of death worldwide [3]. Development of CVD is associated with behavioural risk factors such as smoking, unhealthy diet, obesity and physical inactivity [3]. Geographic variation has been reported for AMI, [4, 5] stroke, [6, 7] and AF [8, 9].

The Danish administrative registers with georeferenced and linkable data on the entire population offer a unique setting for disease mapping studies. In two previous studies, we found geographic variation in AMI [4] and AF, [9] respectively, based on Danish register data. These studies provided detailed analyses of single diseases, including e.g., neighbourhood characteristics [4] and assessment of increasing social inequality in AF over time [9]. In the present study, we aimed specifically at comparing the geographic patterns in AMI, stroke, and AF to identify similarities and differences between the diseases. The geographic pattern in stroke has not previously been evaluated in Denmark.

Compared to our two previous single-disease mapping studies, the present study was based on multivariate modelling with inclusion of a multivariate conditionally autoregressive (MCAR) structure [10] modelled by linear models of co-regionalization [11]. This approach to joint modelling and mapping of multiple diseases also provides estimates of correlation between diseases.

A Finnish study used joint modelling of AMI and stroke to assess similarities between their geographic patterns, [12] but to our knowledge, the geographic patterns in AMI, stroke and AF have not previously been simultaneously modelled and studied.

The objective of this study was to compare the geographic patterns in incident AMI, stroke and AF using nationwide, population-based Danish register data and joint modelling. The analyses were adjusted for age, sex, and income as an indicator of lifestyle and health behaviour.

## Methods

### Study design and population

The study was based on prospectively collected data from nationwide, population-based Danish registers. Registers were linked using the unique personal identification number assigned to each Danish resident at birth or immigration [13].

Three separate study populations were established: one for each of the three considered diseases AMI, stroke and AF. For example, the AMI study population included adults aged ≥ 30 years living in Denmark 1 January 2014 with no previous AMI. In this population, follow-up ended at incident AMI, emigration, death or 31 December 2015, whatever came first. The stroke study population and the AF study population were established in similar ways. The three study populations were restricted to adults aged ≥ 30 years since the diseases are rare in young people. Moreover, at age 30 years, most people will have completed their education and income may serve as an indicator of lifestyle and health behaviour.

For each individual in the study populations, information on date of birth, sex, and residential municipality were available from population registers at Statistics Denmark. Residential municipality was assigned 1 January each year. Based on date of birth, age was calculated as a time-varying variable and grouped into age groups (30–59, 60–69, 70–79, 80 +) based on the distribution of the outcome (Table 1). Annual information on equivalized disposable household income was available from income registers at Statistics Denmark (assigned 31 December each year) and grouped into Low (lowest 20%), Medium (middle 60%) and High (highest 20%) based on age-specific quintiles.

**Table 1:** Characteristics of the acute myocardial infarction (AMI), stroke and atrial fibrillation (AF) cohorts

	AMI			Stroke			AF		
	Cases	PY	IR	Cases	PY	IR	Cases	PY	IR
Total	18,349	6,868,414	26.7	28,006	6,817,459	41.1	39,040	6,790,503	57.5
Sex									
Female	6,791	3,561,742	19.1	13,391	3,506,561	38.2	17,754	3,506,072	50.6
Male	11,558	3,306,672	35.0	14,615	3,310,898	44.1	21,286	3,284,431	64.8
Age									
30–59	4,895	4,256,183	11.5	5,775	4,238,191	13.6	4,805	4,256,227	11.3
60–69	4,470	1,321,559	33.8	5,991	1,313,460	45.6	8,412	1,312,313	64.1
70–79	4,595	859,414	53.5	7,676	848,137	90.5	12,345	831,006	148.6
80 +	4,389	431,258	101.8	8,564	416,943	205.4	13,478	390,956	344.7
Income									
Low	4,572	1,367,521	33.4	6,991	1,357,075	51.5	8,600	1,351,987	63.6
Medium	11,017	4,124,208	26.7	16,448	4,093,957	40.2	23,295	4,077,683	57.1
High	2,760	1,376,685	20.0	4,567	1,366,427	33.4	7,145	1,360,833	52.5

*AMI* acute myocardial infarction, *AF* atrial fibrillation, *PY* person-years, *IR* incidence rate (per 10,000 PY)

### Study area

Denmark covers an area of approximately 43,000 km^2^ and the population counted 5.7 million people on January 1st, 2015. The country is divided into 98 municipalities, which serve as the geographical units in this study. The geographical relationship between municipalities was modelled by a symmetric, binary adjacency matrix (98 × 98) based on municipality borders (i.e. a non-diagonal matrix entry was 1 if two municipalities share a common border and 0 otherwise. Diagonal entries were 0). Seven municipalities were islands with no adjacent municipalities. In the matrix, they were linked to the municipality to which they were connected by ferry or bridge (shown on map in Additional file 1: Figure S1).

### Outcome

Incident AMI, stroke, and AF cases were identified in the Danish National Patient Register (NPR) [14] and in the Danish Register of Causes of Death (RCD) [15]. The International Classification of Disease, 8th revision (ICD-8) was used in 1977 (start of NPR)-1993 and 10th revision (ICD-10) was used in 1994–2015 (AMI: ICD-8 code 410, ICD-10 code I21; stroke: ICD-8 codes 430, 431, 433, 434, 436, ICD-10 codes I60, I61, I63, I649; AF: ICD-8 codes 42,793, 42,794, ICD-10 code I48). Both primary and secondary diagnoses as well as underlying and contributing causes of death were considered. To identify incident cases of AMI, stroke and AF, individuals with a diagnosis before 2014 were excluded from the corresponding study population. For example, AMI cases before 2014 were excluded from the AMI study population.

Previous validation studies on diagnoses in NPR have found positive predictive values (PPV) ≥ 92% for incident AMI and AF [16], whereas PPV = 69.3% has been reported for stroke [17].

### Statistical analysis

A multivariate Poisson model including a disease-specific random effect of municipality was used to simultaneously model the three diseases AMI, stroke and AF.

Let *Y*_*ij*_ denote the number of cases of disease *j* in municipality *i*, *i* = 1,…, 98, *j* = 1,2,3. In each study population, person-years at risk were calculated within sex, age and income groups in each municipality. Let ${T}_{ij}^{k}$, *i* = 1,…, 98, *j* = 1,2,3, *k* = 1,…, 24, denote the person-years at risk in study population *j* in municipality *i* within combination *k* of sex, age and income groups. Then the expected number of cases of disease *j* in municipality *i* is

$${E}_{ij}=\sum_{k=1}^{24}{R}_{j}^{k}{T}_{ij}^{k}, i=1,\dots , 98, j=\mathrm{1,2},3,$$

where ${R}_{j}^{k}$ is the calculated national incidence rate (IR) of disease *j* within combination *k* of sex, age and income groups.

Now, the multivariate Poisson model is given by

$${Y}_{ij}\sim \mathrm{Poisson}({E}_{ij}\mathrm{exp}\left({\beta }_{j}+ {\varphi }_{ij}\right)), i=1, \dots , 98, j=\mathrm{1,2},3,$$

where ${\beta }_{j}$ is a disease-specific intercept and ${\varphi }_{ij}$ is a disease-specific random effect of municipality. The *Y*’s are assumed to be independent given the random effects. The random component $\boldsymbol{\varphi }=({\boldsymbol{\varphi }}_{1},{\boldsymbol{\varphi }}_{2},{\boldsymbol{\varphi }}_{3}){^{\prime}}$, $j=\mathrm{1,2},3$, where ${\boldsymbol{\varphi }}_{j}=({\varphi }_{1j}, \dots , {\varphi }_{98j}){^{\prime}}$, $j=\mathrm{1,2},3$, was modelled by a multivariate conditionally autoregressive (MCAR) structure, [10] and included to account for residual spatial correlation within disease as well as residual correlation between diseases. The spatial correlation structure was based on the adjacency matrix described in the study area section.

The MCAR structure was modelled using linear models of co-regionalization [11]. The approach is based on writing the random component $\boldsymbol{\varphi }$ as a linear combination of latent spatial processes ${{\varvec{u}}}_{j}=({u}_{1j},{\dots , u}_{98j}){^{\prime}}$, $j=\mathrm{1,2},3$. Thus, let

$$\boldsymbol{\varphi }=\left({\varvec{A}} \otimes {{\varvec{I}}}_{98x98}\right){\varvec{u}},$$

where ${\varvec{u}}=({{\varvec{u}}}_{1},{{\varvec{u}}}_{2}, {{\varvec{u}}}_{3}){^{\prime}}$ and ${\varvec{A}}$ is the 3 × 3 lower triangular matrix uniquely determined as the Cholesky decomposition of a matrix $\boldsymbol{\Sigma }={\varvec{A}}{\varvec{A}}\boldsymbol{^{\prime}}$. Now, the covariance structure within $\boldsymbol{\varphi }$, i.e. within and between diseases, depends on the modelling of ${\varvec{u}}$.

Each process ${{\varvec{u}}}_{j}$, $j=\mathrm{1,2},3$, was modelled by a conditional autoregressive (CAR) model. In the most complex model considered, the processes ${{\varvec{u}}}_{1},{{\varvec{u}}}_{2},{{\varvec{u}}}_{3}$ were assumed independent with ${{\varvec{u}}}_{j}$ modelled by a Leroux CAR model [18] with spatial correlation parameter ${\rho }_{j}$, $j=\mathrm{1,2},3$ (Model 1). This allows for different spatial structures within each of the three diseases and correlation between diseases is municipality specific (details elsewhere [11, 19]). This was simplified by assuming a common correlation parameter $\rho$ for all ${{\varvec{u}}}_{j}$, $j=\mathrm{1,2},3$ (Model 2). In that case, the same spatial structure is assumed within all diseases and correlation between diseases is not municipality specific (equivalent to the separable model suggested by Gelfand and Vounatsou [10]). A simple model assuming no correlation between diseases were also considered (Model 3). This corresponds to standard, univariate modelling of each disease. In this model, disease-specific spatial correlation parameters were assumed.

Bayesian inference was based on Markov chain Monte Carlo (MCMC) methods using WinBUGS [20] and the approach described in detail by MacNab [19]. Instead of assigning a prior to ${\varvec{A}}$, priors were assigned to the elements of $\boldsymbol{\Sigma }$, and ${\varvec{A}}$ was determined by the one-to-one relationship between $\boldsymbol{\Sigma }$ and ${\varvec{A}}$. The matrix $\boldsymbol{\Sigma }$ could be considered a covariance matrix, and uniform (0,1) priors were assigned to the correlation parameters, whereas uniform (0,10) priors were assigned to the standard deviation parameters. The spatial correlation parameter of the Leroux model was assigned a uniform (0,1) prior. The intercept term was assigned a weakly informative Gaussian prior with mean zero and precision 0.1 to improve convergence. In a sensitivity analysis, the prior on the standard deviation parameters was changed to uniform (0,100).

Posterior inference was based on a total of 10,000 samples generated from two chains with different initial values. The first 10,000 samples were discharged as burn-in, and the chains were thinned to every 100th sample to reduce autocorrelation. Convergence was evaluated by visual inspection of trace and density plots, autocorrelation plots, and Geweke diagnostics [21]. Effective sample size was also considered.

Point estimates are reported as the median of the posterior distribution together with 95% credible intervals (CI). Models were compared using the deviance information criterion (DIC) [22].

Based on the multivariate model, estimated municipality-specific standardized incidence rates (SIR) of AMI, stroke and AF, respectively, where calculated as ${\widehat{Y}}_{ij}/{E}_{ij}$, *i* = 1, …, 98, *j* = 1,2,3, where ${\widehat{Y}}_{ij}$ is the estimated number of cases of disease *j* in municipality *i*. The estimated SIRs were mapped to display a smoothed map of municipality-specific SIRs for each disease.

Conditional correlation between diseases at collocation were derived from the 3 × 3 covariance matrix $\boldsymbol{\Sigma }$ [19].

## Results

The study included 3,501,382 individuals contributing 6,868,414 person-years at risk of AMI, 3,477,096 individuals contributing 6,817,459 person-years at risk of stroke and 3,467,377 individuals contributing 6,790,503 person-years at risk of AF. In total, 18,349 incident cases of AMI occurred during the study period, resulting in an IR of 26.7 per 10,000 person-years, 28,006 incident cases of stroke occurred, resulting in an IR of 41.1 per 10,000 person-years, and 39,040 incident cases of AF occurred, resulting in an IR of 57.5 per 10,000 person-years (Table 1). For all three diseases, men had the highest IR, and the IRs increased with age, whereas they decreased with increasing income.

An overview of the data management process is given in the data flow diagram in Additional file 2: Figure S2.

Fitting the three considered models, Model 2 provided the best fit to data (DIC_Model2_ = 2619 compared to DIC_Model1_ = 2623 and DIC_Model3_ = 2627). Therefore, the results from Model 2 are presented. The results from Model 1 were very similar, whereas Model 3 produced slightly different results (Additional file 3: Figures S3 and S4). Model 3, however, did not account for pairwise correlation between diseases and provided the poorest fit to data (DIC_Model3_ = 2627).

**Fig. 1:**
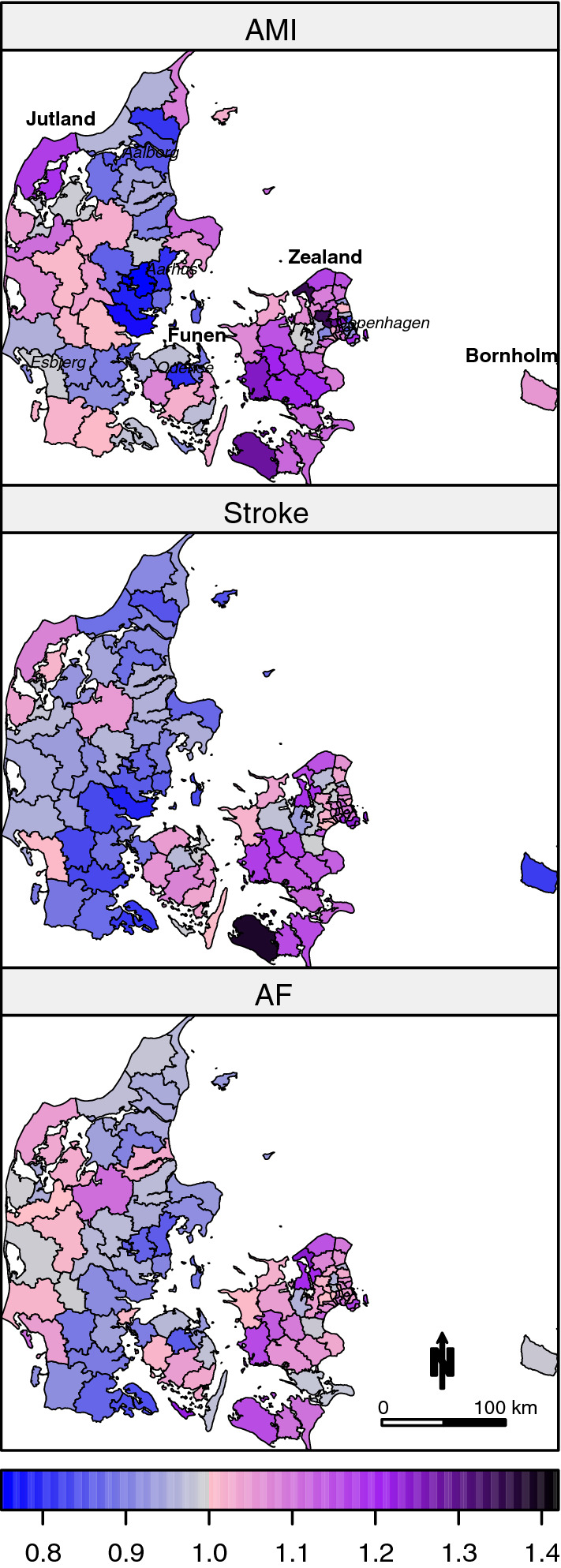
Smoothed maps of municipality-specific estimated standardized incidence rates (SIR) of acute myocardial infarction (AMI), stroke and atrial fibrillation (AF). An SIR value close to 1 indicates that the municipality-specific IR is similar to the overall national IR (sex, age and income adjusted), whereas SIR values above or below 1 indicate municipality-specific IRs above or below the national IR. Names in italics indicate location of the five largest cities, including the capital Copenhagen

The estimates from Model 2 are mapped in Fig. 1 to display a smoothed map of municipality-specific SIRs for each disease. An SIR value close to 1 indicates that the municipality-specific IR is similar to the overall national IR (sex, age and income adjusted), whereas SIR values above or below 1 indicate municipality-specific IRs above or below the national IR.

In general, the SIRs appeared to be highest in the eastern part of the country (Zealand), whereas the eastern part of Jutland appeared to have the lowest SIRs. AF showed the least variation between municipalities with SIRs in general closer to 1 than SIRs of AMI and stroke. Municipality-specific AMI SIRs ranged from 0.76 to 1.35 corresponding to municipality-specific IRs ranging from 24% below to 35% above the national IR, stroke SIRs ranged from 0.79 to 1.38 corresponding to IRs ranging from 21% below to 37% above the national IR, and AF SIRs ranged from 0.85 to 1.24 corresponding to IRs ranging from 15% below to 24% above the national IR.

**Fig. 2:**
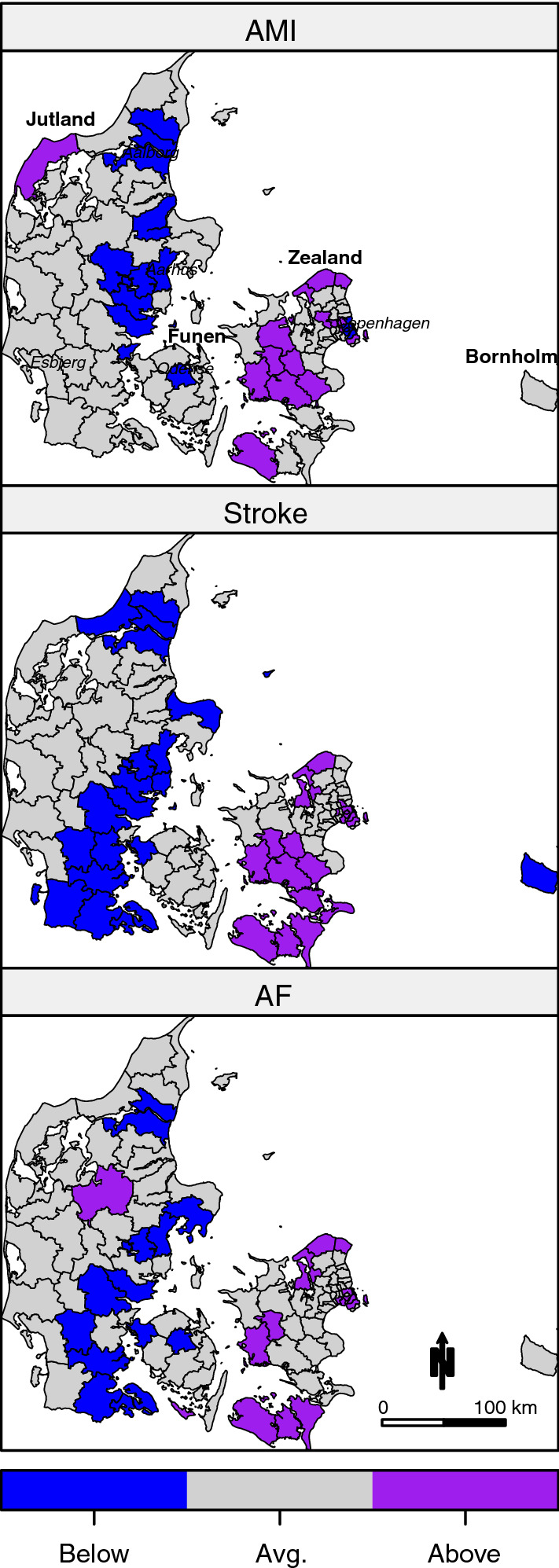
Municipalities with estimated standardized incidence rates (SIR) of acute myocardial infarction (AMI), stroke and atrial fibrillation (AF) significantly above or below 1. These maps illustrate municipalities with significantly high or low risk of disease compared to the national average after accounting for age, sex and income. Names in italics indicate location of the five largest cities, including the capital Copenhagen

The municipalities with an estimated SIR significantly above or below 1 are displayed in Fig. 2. These maps illustrate municipalities with significantly high or low risk of disease compared to the national average after accounting for age, sex and income. AMI SIRs were below 1 in four out of the five largest cities, including Copenhagen. In contrast, SIRs of both stroke and AF were above 1 in Copenhagen. In the middle part of Zealand, SIR above 1 was not as widespread for AF as for AMI and stroke. Both stroke and AF had SIRs below 1 in southern Jutland, which was not found for AMI, whereas the pattern in eastern/northern Jutland tended to be similar (SIR < 1) for all three diseases. In addition, a high-risk area unique to AMI, and one unique to AF were seen in Jutland.

Based on the model, conditional correlation between diseases at collocation were 0.47 (0.22; 0.67) between AMI and stroke, 0.42 (0.14; 0.64) between AMI and AF, and 0.65 (0.42; 0.81) between stroke and AF. The spatial correlation between municipalities was 0.67 (0.28; 0.98).

Results were not sensitive to changes in the prior distributions.

## Discussion

This study evaluated and compared the geographic patterns in incident AMI, stroke and AF using nationwide, population-based Danish register data. Multiple-disease mapping was used to simultaneously model the three diseases. In all diseases, geographic variation with clusters of high or low risk of disease compared to the national average after accounting for age, sex and income was seen. The geographic patterns displayed overall similarities between the diseases, with stroke and AF having the strongest resemblances. Stroke and AF also exhibited the strongest correlation between diseases. The most notable difference between diseases was observed in Copenhagen, which had significantly high risk of both stroke and AF, but significantly low risk of AMI.

### Comparison with other studies

Few previous studies did multiple-disease mapping of CVD. In Finland, a marked part of the geographic variation in AMI and stroke risk was found to be disease specific [12]. The study, however, used a different modelling approach than the present study, and the results are not directly comparable. A study from Scotland used the same modelling approach as the present study, but considered less specific disease outcomes: cerebrovascular disease (incl. stroke), coronary heart disease (incl. AMI and AF) and also respiratory disease [23]. Furthermore, they also considered changes over time. In the United States (US), a study on male physicians found significantly increased risk of stroke associated with the south-eastern region usually referred to as the “stroke belt”, whereas the risk of AMI in this region was significantly decreased compared to the rest of the US [6]. Another study found no systematic pattern in AF hospitalization rates in the stroke belt states [8]. These US results in a way match the present results on AMI and stroke in Copenhagen, and on stroke and AF on Zealand. The very different geographic scales, however, make comparisons between studies difficult.

Overall, the geographic patterns in AMI and AF match the results of previous Danish single-disease studies [4, 9]. The geographic pattern in stroke has not previously been evaluated in Denmark.

### Interpretation of results

The multivariate models with correlation between diseases provided better fits to data than the model with no correlation between diseases. This suggests the presence of significant correlation between diseases. Stroke and AF displayed the strongest correlation and geographic resemblances. This may reflect the fact that AF is a strong risk factor for stroke, [2] whereas the association (which may work in both directions) between AF and AMI is weaker [1, 24].

In general, finding geographic patterns in incident disease may indicate clustering in the underlying risk of developing disease. This may primarily point towards environmental risk factors, including those related to lifestyle and health behavior which may vary geographically. In the present study, the observed similarities between the geographic patterns suggest that AMI, stroke and AF share some common risk factors beyond those accounted for, i.e. age, sex and income. This was to some extent expected since data on behavioural risk factors such as smoking, unhealthy diet and physical inactivity, which are known to be of importance for the development of all CVDs, were not available. Instead, information on income, in addition to age and sex, was included to account for at least some of the variation in lifestyle and health behaviour but may not capture it all. Compared to AMI and stroke, AF showed the least variation between municipalities, suggesting the weakest association with environmental factors.

The differences between the geographic patterns were more subtle than the similarities but may still indicate some basic differences in risk factors (or their effect) between AMI, stroke and AF. For example, in Copenhagen, where a low-risk cluster of AMI displayed high risk of both stroke and AF. This suggests that stroke and AF are associated with some underlying risk factors beyond those associated with AMI and that these cluster in Copenhagen. AMI is primarily caused by atherosclerosis, and the INTERHEART study [25] showed that nine easily measured risk factors (Abnormal lipids, smoking, hypertension, diabetes, abdominal obesity, psychosocial factors, lack of consumption of fruits, vegetables, and alcohol, and lack of regular physical activity) are associated with more than 90% of the risk of an AMI. Stroke is strongly related to hypertension and AF and includes both ischemic and hemorrhagic event. AF is an arrhythmia and might lead to heart failure and stroke; however, the etiology is more complex and not only related to atherosclerosis but also metabolic disorders, high alcohol intake, structural heart disease etc.

Competing risks may also contribute to the observed geographic differences, since some patients may die from one disease (most likely AMI or stroke) before having any of the two other diseases. For example, in the middle part of Zealand, where the high-risk cluster of AF seemed to be smaller than those of AMI and stroke. This may reflect geographic differences in mortality, which has been shown to exist for AMI, [26] but has not been evaluated for stroke in a Danish context. Furthermore, especially cases of AF, the least severe condition of the three, may be undiagnosed. The Danish healthcare system is available to all Danish citizens free of charge but still, the actual use of healthcare may vary across the country. This was seen in a population of AMI patients with large variation in use of general practitioner the year before diagnosis [27]. This may cause geographic differences in the proportion of undiagnosed patients, which influence the geographic pattern. In the analysis, no distinction was made between sub-diagnoses within each disease (e.g. haemorrhage stroke and ischemic stroke), although their aetiology and risk factors may vary. In particular, AF is not associated with haemorrhage stroke (constituting around 17% of all strokes [17]), suggesting that this type of stroke may be relatively more frequent in areas with high risk of stroke but not of AF.

Overall, the results indicate that some areas carry a particularly heavy disease burden from the considered CVDs. This should be taken into consideration in planning of prevention strategies and allocation of resources.

### Strengths and limitations

A major strength of this study is the use of nationwide, population-based data from administrative registers that can be linked at individual level and provide complete information on hospitalizations and deaths. A limitation in the use of these data is the potential risk of misclassification of disease within the registers, as well as underreporting of disease since individuals only attending general practitioner or not seeking medical attention at all are not registered in NPR. In this study with focus on geographic variation, both misclassification and underreporting are mainly a concern if they depend on geographic location.

The combination of NPR and RCD data has been found to provide valid information on AMI incidence with only minor misclassification and undereporting [28]. AF is also a valid diagnosis in NPR, [29] but underreporting has not been evaluated and unrecognized AF may occur, as discussed above. Within the stroke diagnosis, 30% misclassification has been reported [17]. The degree of misclassification depended on hospital department with inpatient clinics and especially specialised units having the least misclassification (e.g. neurology 12% misclassification). The availability of specialized units may vary across the country, causing systematic geographic variation in misclassification. On the other hand, some authors have suggested the extensive use of neuroimaging in all parts of the country is likely to attenuate the difference in diagnostics between urban and rural settings [30]. All in all, however, some effect of misclassification on the geographic pattern in stroke cannot be ruled out. Regarding underreporting, a study found that < 5% of the stroke events in a Copenhagen-area sub-population were nonhospitalized and nonfatal, [31] which suggests only minor underreporting in the registers.

All information included in the analysis were available at individual level, but data were aggregated at municipality level for computational reasons in order to ensure manageable computing times. In general, the results may be sensitive to the choice of geographical unit, also known as the modifiable areal unit problem (MAUP) [32]. In this study, the chosen scale enabled identification of geographic clusters of a certain size, but additional small-scale clustering may exist.

## Conclusions

Using multiple-disease mapping, this study adds to the results of previous studies by enabling joint evaluation and comparison of the geographic patterns in incident AMI, stroke and AF. The simultaneous mapping of diseases displayed similarities and differences in occurrence that are non-assessable in traditional single-disease mapping studies, and therefore provided enhanced information compared to those. Moreover, observing similarities between the geographic patterns strengthen the indication of clustering in the underlying risk of disease.

In conclusion, the present study suggested that AMI, stroke and AF share some, but not all environmental risk factors after accounting for age, sex and income (as an indicator of lifestyle and health behaviour). Identification of specific determinants of the observed geographic patterns was beyond the scope of this study and remains a topic for future research.

## Supplementary Information

**Additional file 1: Figure S1.** Map illustrating the definition of geographical relationship across water.

**Additional file 2: Figure S2.** Data flow diagram illustrating data management for developing the acute myocardial infarction (AMI), stroke and atrial fibrillation (AF) cohorts.

**Additional file 3: Figures S3 and S4.** Smoothed maps and significance of municipality-specific estimated standardized incidence rates (SIR) of acute myocardial infarction (AMI), stroke and atrial fibrillation (AF).

## Data Availability

The data that support the findings of this study are available from the National Health Authority and Statistics Denmark, but restrictions apply to the availability of these data, which were used under license for the current study, and so are not publicly available.

## Abbreviations

AMI: Acute myocardial infraction
AF: Atrial fibrillation
CVD: Cardiovascular disease
NPR: National Patient Register
RCD: Register of Causes of Death
ICD: International Classification of Disease
PPV: Positive predictive value
IR: Incidence rate
SIR: Standardized incidence ratio
MCAR: Multivariate conditionally autoregressive
MCMC: Markov chain Monte Carlo
CI: Credible intervals
DIC: Deviance information criteria
US: United States
MAUP: Modifiable areal unit problem
