# Supplementary material for: Joint mapping of cardiovascular diseases: comparing the geographic patterns in incident acute myocardial infarction, stroke and atrial fibrillation, a Danish register-based cohort study 2014–15
Source: Int J Health Geogr. 2021 Aug 30;20:41. doi: 10.1186/s12942-021-00294-w (PMC8404297; doi:10.1186/s12942-021-00294-w)

**Figure S.1.** Map illustrating the definition of geographical relationship between municipalities across water.


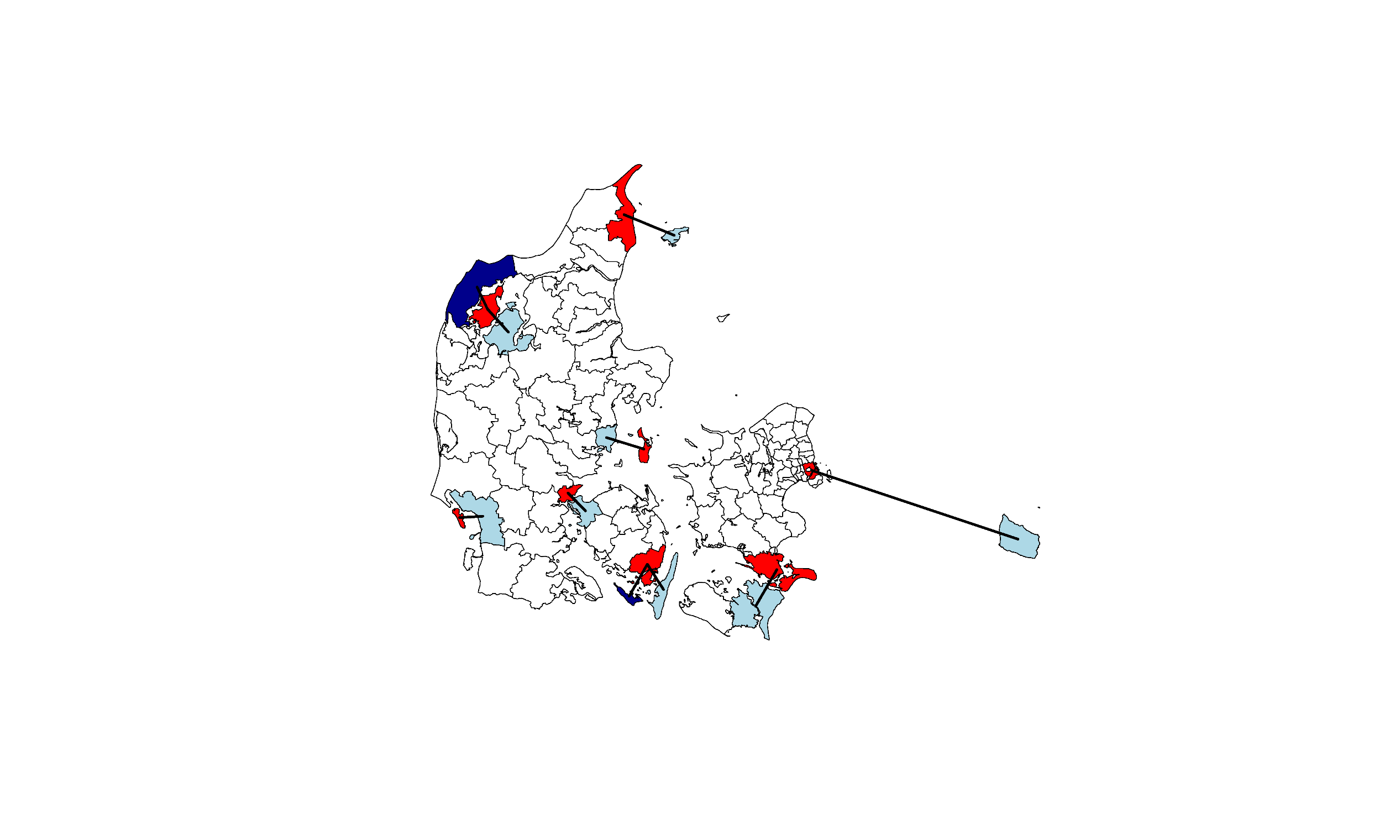

Supplement: Supplementary file 1 — Additional file 1: Figure S1. Map illustrating the definition of geographical relationship across water. [file 12942_2021_294_MOESM1_ESM.docx]
