# Supplementary material for: Joint mapping of cardiovascular diseases: comparing the geographic patterns in incident acute myocardial infarction, stroke and atrial fibrillation, a Danish register-based cohort study 2014–15
Source: Int J Health Geogr. 2021 Aug 30;20:41. doi: 10.1186/s12942-021-00294-w (PMC8404297; doi:10.1186/s12942-021-00294-w)
Supplement: Supplementary file 2 — Additional file 2: Figure S2. Data flow diagram illustrating data management for developing the acute myocardial infarction (AMI), stroke and atrial fibrillation (AF) cohorts. [file 12942_2021_294_MOESM2_ESM.pdf]

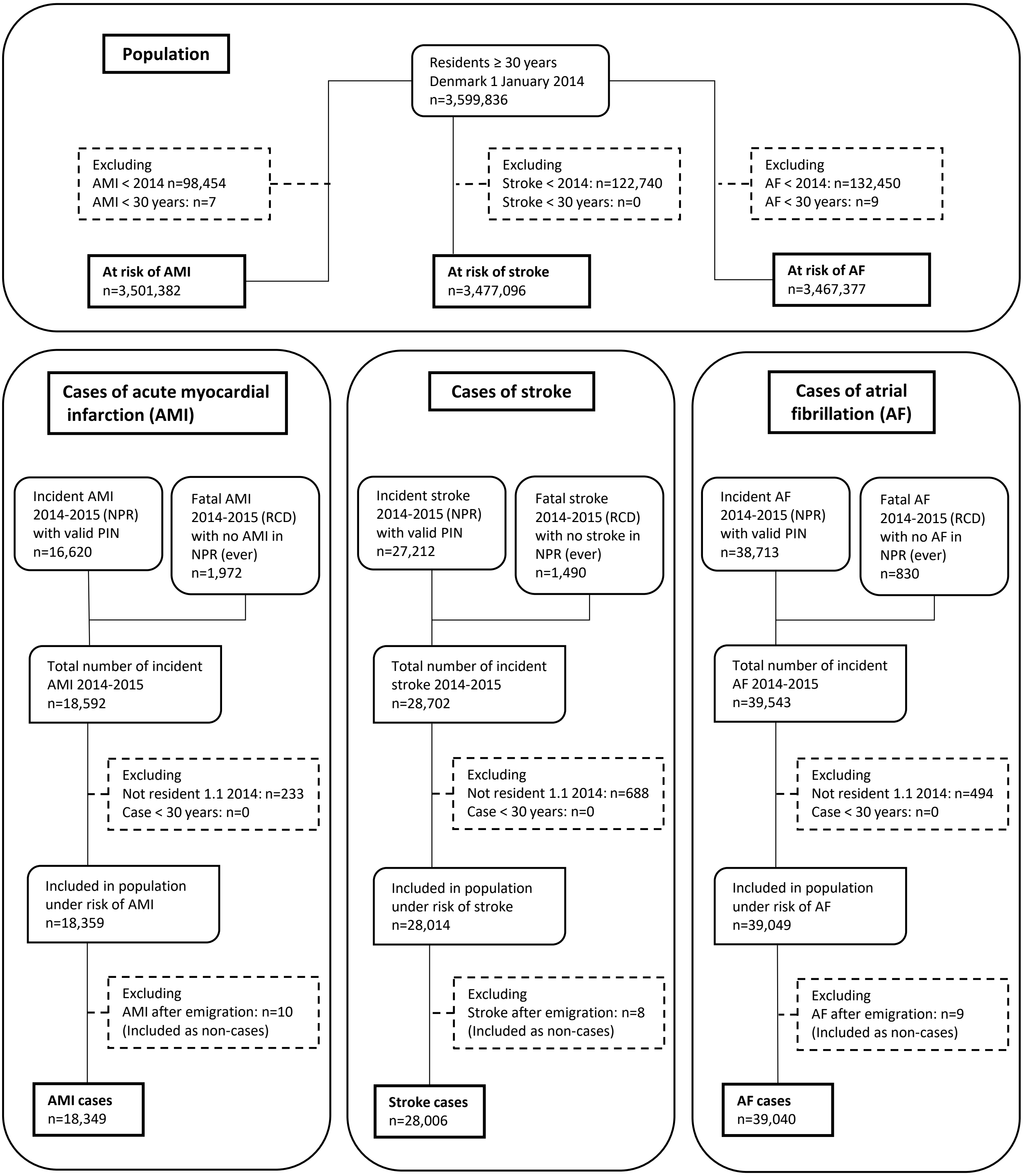

Figure S.1. Data flow diagram illustrating data management for developing the acute myocardial infarction (AMI), stroke and atrial fibrillation (AF) cohorts.
