# Supplementary material for: Joint mapping of cardiovascular diseases: comparing the geographic patterns in incident acute myocardial infarction, stroke and atrial fibrillation, a Danish register-based cohort study 2014–15
Source: Int J Health Geogr. 2021 Aug 30;20:41. doi: 10.1186/s12942-021-00294-w (PMC8404297; doi:10.1186/s12942-021-00294-w)

**Figure S.3**. Smoothed maps (left) and significance (right) of municipality-specific estimated standardized incidence rates (SIR) of acute myocardial infarction (AMI), stroke and atrial fibrillation (AF) based on Model 1. Municipalities with estimated SIR significantly above or below 1 have a significantly high or low risk of disease compared to the national average after accounting for age, sex and income. Names in italics indicate location of the five largest cities, including the capital Copenhagen.


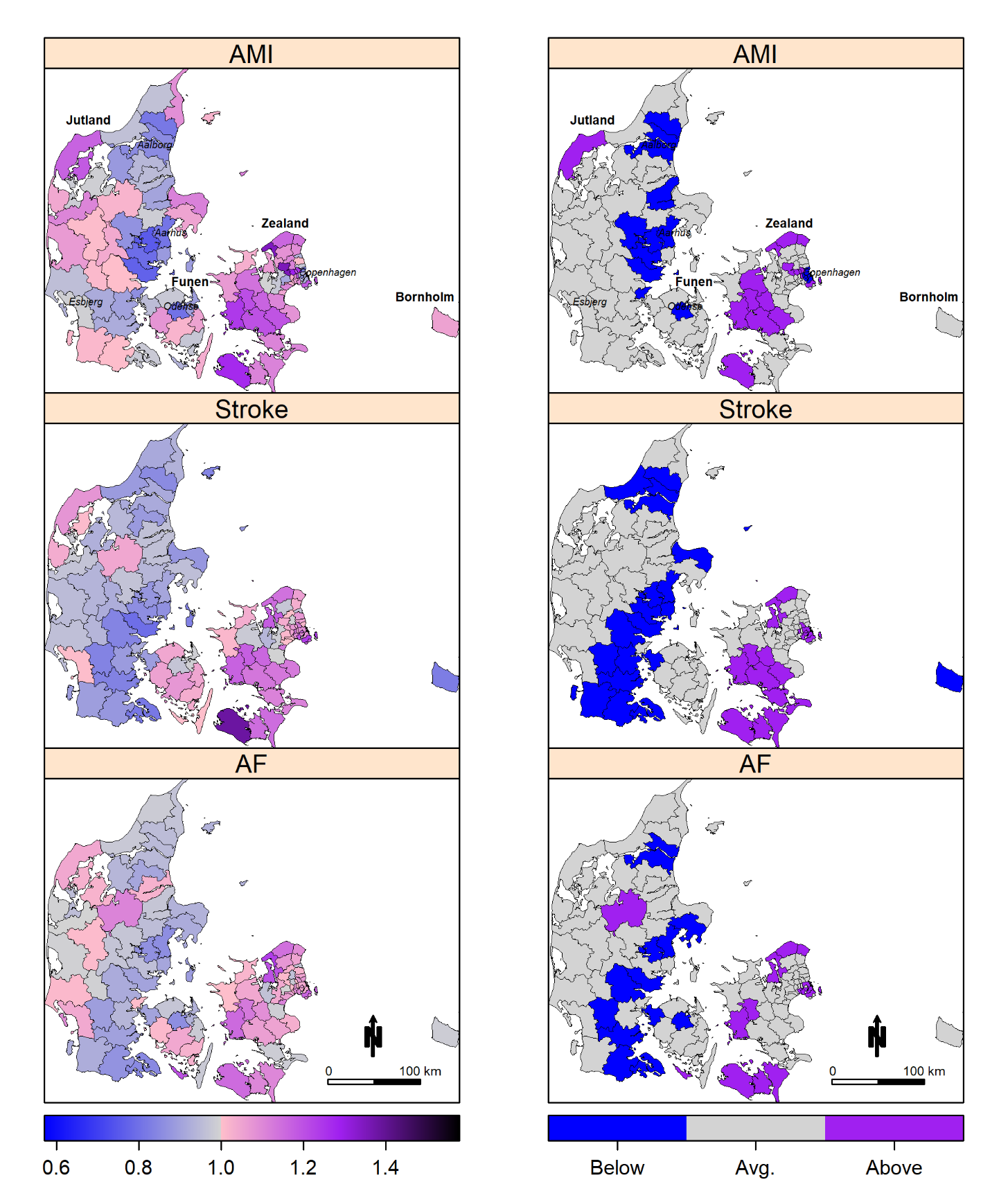


**Figure S.4.** Smoothed maps (left) and significance (right) of municipality-specific estimated standardized incidence rates (SIR) of acute myocardial infarction (AMI), stroke and atrial fibrillation (AF) based on Model 3. Municipalities with estimated SIR significantly above or below 1 have a significantly high or low risk of disease compared to the national average after accounting for age, sex and income. Names in italics indicate location of the five largest cities, including the capital Copenhagen.


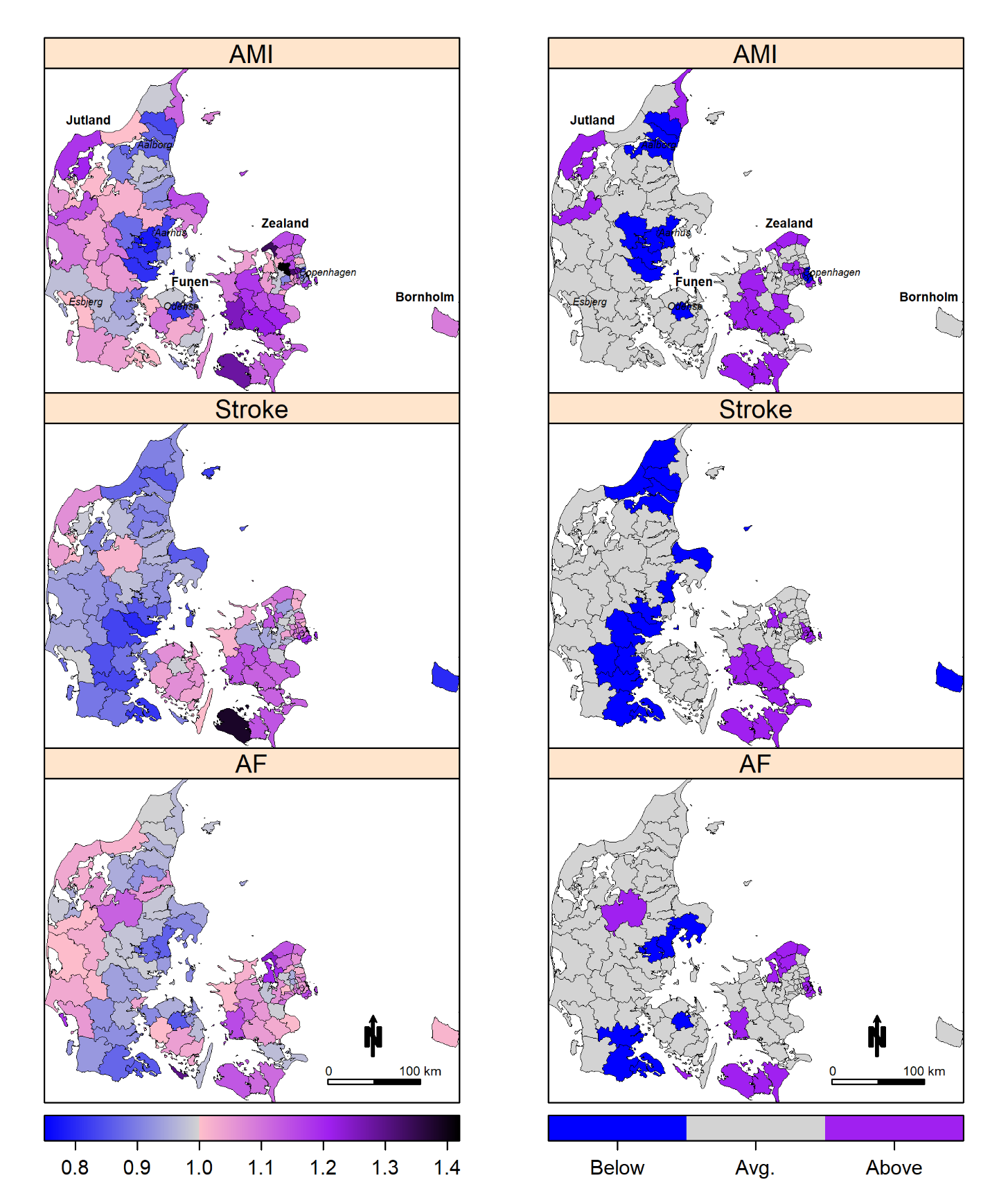

Supplement: Supplementary file 3 — Additional file 3: Figures S3 and S4. Smoothed maps and significance of municipality-specific estimated standardized incidence rates (SIR) of acute myocardial infarction (AMI), stroke and atrial fibrillation (AF). [file 12942_2021_294_MOESM3_ESM.docx]
